# A Genome-Wide Association Study of Rib Number and Thoracolumbar Vertebra Number in a Landrace × Yorkshire Crossbred Pig Population

**DOI:** 10.3390/biology14081068

**Published:** 2025-08-16

**Authors:** Chunyan Bai, Junwen Fei, Xiaoran Zhang, Wuyang Liu, Juan Ke, Changyi Chen, Yu He, Shuang Liang, Boxing Sun, Hao Sun

**Affiliations:** College of Animal Science, Jilin University, Changchun 130062, China; bcy@jlu.edu.cn (C.B.);

**Keywords:** vertebral number, rib number, thoracolumbar vertebrae, GWAS (Genome-Wide Association Study), bone development genes

## Abstract

The number of thoracolumbar vertebrae (NTLV) and the number of ribs (NR) are economically important traits in pigs due to their influence on carcass length and meat yield. Although *VRTN* is an established key gene, it fails to fully account for population-level variation in vertebral count, necessitating a further exploration of its genetic mechanisms. Given the efficacy of crossbred populations in mapping the genetic determinants of phenotypic variation, we analyzed 439 pigs from a Landrace × Yorkshire cross. Genotyping was performed via a 50 K SNP chip. Both NTLV and NR showed high heritability (0.700 and 0.752, respectively), while the number of lumbar vertebrae (NLV) showed limited variation (92.5% of pigs had NLV = 6). Using the BLINK model, four significant loci were identified. The most significant SNP, rs3469762345, located in the intergenic region between *ABCD4* and *VRTN*, corresponds to a previously known QTL. Additionally, three novel variant sites (rs81211244, rs81347323, and rs81416674) were identified within or near the *ALDH7A1*, *PTPRT*, and *PAK1* genes, which are known to play a role in bone development. This study uncovers novel swine candidate genes associated with vertebral and rib number variation, subsequently facilitating targeted research into their molecular mechanisms.

## Simple Summary

The number of vertebrae and ribs in pigs affects their body length and meat yield, making these traits important for pig breeding. In this study, we analyzed the DNA of 439 pigs from a commercial population to find genes related to these traits. Four specific genetic markers were found to be linked to the number of vertebrae and ribs. One of these markers was already known, while the others are newly found and located in genes related to bone development. These findings help scientists better understand how these traits are controlled and may be useful for improving pig breeding through genetic selection.

## 1. Introduction

The thoracolumbar vertebra comprises thoracic vertebrae and lumbar vertebrae in pigs. The number of thoracic vertebrae (NTV) is equal to the number of ribs (NR). The number of thoracolumbar vertebrae (NTLV), particularly rib number, is an economically significant trait in pig production, as it directly impacts carcass quality and meat yield. The NR and NTLV traits in pigs vary within and among populations. Generally, it has been reported that NR ranges from 13 to 17 [1], while NTLV ranges from 19 to 23 [2]. Van Son et al. reported significantly high heritability for both vertebral counts (*h*^2^ = 0.62) and rib numbers (*h*^2^ = 0.78) [3]. Due to their high heritability and commercial relevance, identifying genes regulating vertebral development is crucial for breeding swine with longer carcasses and improved lean yield.

Genome-wide association studies (GWASs) are classical methods used to identify genetic loci underlying phenotypic variation. The pigQTL database [4] compiles numerous GWASs on economically important traits in swine, including five studies investigating rib number variation [1,5,6,7,8]. These studies consistently identified chromosome 7, particularly the *VRTN* gene, as a key genetic determinant of rib number variation. Additionally, independent studies not cataloged in the pigQTL database likewise identified *VRTN* on chromosome 7 as a key regulator of rib number variation [9,10,11]. While existing research confirms *VRTN* as a pivotal gene, rib number variation is unlikely to be governed solely by this locus. Novel genetic loci influencing this trait have been identified on chromosomes 1, 4, and 6 [5,7].

Crossbred populations are ideal for GWASs due to their enriched genetic and phenotypic diversity compared to purebred cohorts. Significant phenotypic variation in rib number was observed within a commercial crossbred population (Landrace × Yorkshire ancestry) at a local breeding farm. To our knowledge, no studies have characterized the genetic architecture underlying rib number variation in Landrace–Yorkshire hybrids. Given heterogeneity in genetic architectures across populations, investigations utilizing novel cohorts may reveal previously unidentified associations. Therefore, it is necessary to conduct association studies across more populations to provide a broader perspective for investigating the genetic and phenotypic variations in these traits. To elucidate the genetic architecture of vertebral and rib number variation in this population, we performed a genome-wide association study (GWAS).

## 2. Materials and Methods

### 2.1. Phenotypic and Genotypic Data Preparation

Phenotypic measurements of NTLV and NR were collected from 439 randomly selected carcasses with similar weight derived from a Landrace × Yorkshire rotational crossbred commercial population. These pigs were reared at a local swine breeding farm in Tongyu, Jilin Province, China. Two trained technicians quantified NTLV and NR via manual palpation on left hemilateral carcasses post mid-thoracic sagittal bisection. Genomic DNA was extracted from *longissimus dorsi* muscle samples using the DC112 commercial kit (Vazyme Biotech, Nanjing, China). All 439 animals were genotyped using a 50 K chip. Autosomal SNPs with a call rate of more than 90% were retained. The missing genotypes were imputed using BEAGLE (version 5.4; beagle. 29 October 2024. c8e.jar) [12]. Finally, a total of 48,329 SNPs with a minor allele frequency (MAF) more than 1% were retained for subsequent analysis.

### 2.2. Genetic Variance Estimation

Genetic variance was estimated using Hiblup (V1.5.0) software [13] based on a single trait model with its default parameters. The model was defined as *y* = *u* + *a* + *e*, where *y* is the phenotype; *u* is the mean term; *a* is the additive genetic effect; *e* is the residual effect. The additive genetic effect (*a*) and residual effect (*e*) both follow multivariate normal distributions: $a\sim N(0,G\sigma_{a}^{2})$ and $e\sim N(0,I\sigma_{e}^{2})$, where G is the kinship matrix, $\sigma_{a}^{2}$ is the genetic variance, I is an identity matrix, and $\sigma_{e}^{2}$ is the residual variance. The heritability ($h^{2}$) was calculated as $h^{2}=\sigma_{a}^{2}$/($\sigma_{a}^{2}$+$\sigma_{e}^{2}$).

### 2.3. Genome-Wide Association Studies

Genome-wide association studies were conducted using two complementary approaches: (1) a classical mixed linear model (MLM) implemented in GCTA [14] and (2) the Bayesian-information and Linkage-disequilibrium Iterated Conditional Key (BLINK) model via GAPIT3 [15]. The first three principal components were included as covariates in the analysis to control for population structure.

Significance thresholds were determined using the Bonferroni correction method (calculated as −log10(α/N), where α = 0.05, and N = 48,329), resulting in a genome-wide significance level of 5.985. Significant loci were functionally annotated using the Ensembl genome browser [16] and PigQTL database (V56) [4].

## 3. Results and Discussion

### 3.1. Descriptive Statistics and Genetic Parameters for the Phenotype

**Figure 1:**
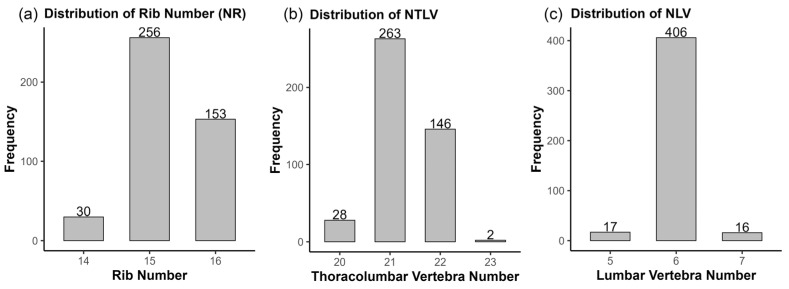
Frequency distributions of (**a**) rib number (NR), (**b**) thoracolumbar vertebrae (NTLV), and (**c**) lumbar vertebrae (NLV) in study population.

Table 1 summarizes the descriptive statistics and genetic parameters for the phenotype of NTLV, NR, and the number of lumbar vertebrae (NLV = NTLV − NR) in the study population. The mean values for NTLV, NR, and NLV were 21.3, 15.3, and 6.0, with ranges of 20~23, 14~16, and 5~7, respectively. The phenotypic distributions of these traits in the studied population are illustrated in Figure 1a–c. Substantial variation was observed for NR (Figure 1a), while NLV exhibited limited polymorphism (Figure 1c). Among the 439 analyzed individuals, 406 (92.5%) displayed NLV = 6, with only 17 (3.8%) and 16 (3.6%) individuals exhibiting NLV = 5 and NLV = 7, respectively. This constrained phenotypic variation in NLV suggests that the observed differences in NTLV are primarily driven by variation in NR rather than lumbar vertebral count.

The heritability (*h*^2^) estimates revealed strong genetic control for NTLV (0.700) and NR (0.752) (Table 1). In contrast, the heritability estimate for lumbar vertebra number (NLV) was near zero (*h*^2^ = 0.017), which is likely attributable to the limited phenotypic variation in this trait observed in the studied population.

The *h*^2^ estimates of NTLV and NR in our study are generally higher than those in previous studies. In Rohrer et al.’s research, the *h*^2^ estimates of NTLV and NR were 0.24 and 0.16 [2], respectively. Liu et al. reported that the *h*^2^ estimate of NR was 0.323 [5]. In addition, in a study by Niu et al., the *h*^2^ estimate of NR was 0.59 [7]. Our study observed higher heritability estimates for both the NR and NTLV traits than were reported in the previous literature. One possible reason for this is the use of a crossbred pig population, which is likely to exhibit greater genetic diversity than purebred cohorts. Enhanced genetic diversity can increase additive genetic variance, thereby elevating the estimates.

### 3.2. Results of Genome-Wide Association Studies

**Figure 2:**
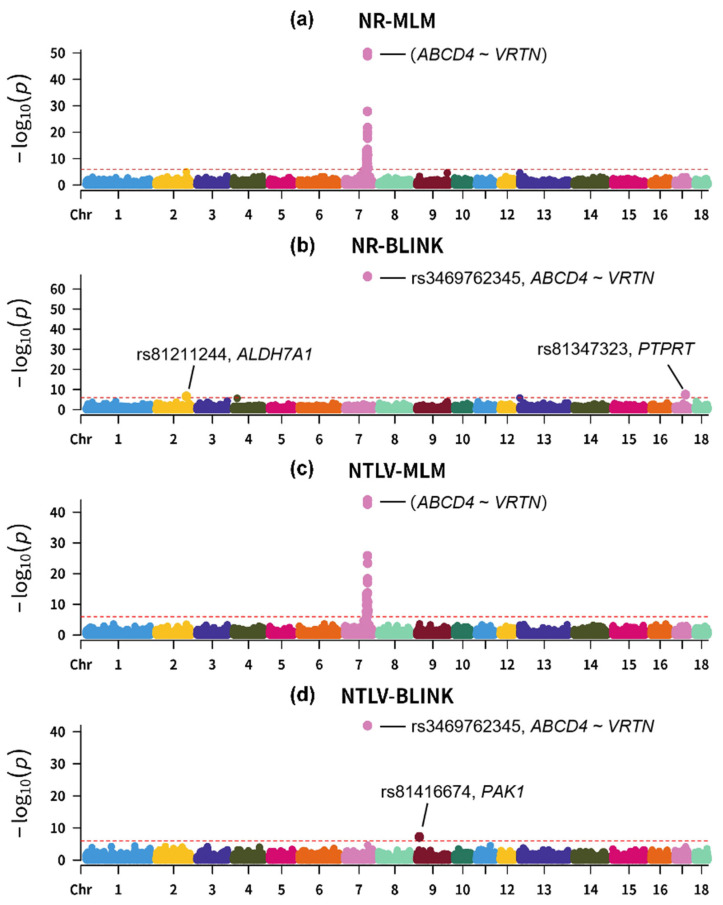
(**a**) Manhattan plot for rib number (NR) under the MLM framework; (**b**) Manhattan plot for rib number (NR) under the BLINK framework; (**c**) Manhattan plot for thoracolumbar vertebra number (NTLV) under the MLM framework; (**d**) Manhattan plot for thoracolumbar vertebra number (NTLV) under the BLINK framework. The red horizontal line indicates the genome-wide significance threshold, set at a −log_10_(*p*) value of 5.985.

A 3D PCA plot is shown in Figure S1. Figure 2 presents the Manhattan plots for the GWAS results of the NTLV and NR traits based on the MLM and BLINK model (Figure S2). Based on the MLM analysis, this study identified 35 significant SNPs (Table S1) associated with NR and 38 significant SNPs (Table S2) associated with NTLV, all located on chromosome 7. Notably, SNPs that showed genome-wide significant associations with the NR trait were also significantly associated with the NTLV trait.

Among these significant SNPs, three SNPs exhibited exceptionally strong associations (*p* < 1 × 10^−40^) with both the NR and NTLV traits: chr7:97575068 (rs701714758), chr7:97595573 (rs3469762345), and chr7:97614602 (rs709317845). According to the Ensembl database, the SNP rs1113960993 was located in the intron region of the *ABCD4* gene, and the SNPs rs3469762345 and rs709317845 were located in the intergenic region between *ABCD4* (chr7:97568208-97585655) and *VRTN* (chr7:97614707-97624273). The above three SNPs (rs701714758, rs3469762345, and rs709317845) are cataloged in the Pig QTLdb (release 56). SNP rs701714758 is annotated in two independent studies: one associating it with teat number [17], the other with body circumference [18]. SNP rs3469762345 was reported in five independent studies: two studies associated it with NR [12,13], one with vertebra number [15], one with teat number [16], and one with body circumference [17]. SNP rs709317845 was also reported in five independent studies: two studies associated it with thoracic vertebrae number [19,20], one with rib number [5], one with teat number, and one with longissimus muscle depth [21].

Based on the BLINK analysis, this study identified three significant SNPs associated with NR and two significant SNPs associated with NTLV (Table S3). Strikingly, only one SNP on chromosome 7 (chr7:97595573, rs3469762345) showed pleiotropic effects, reaching significance for both traits. Compared to the MLM approach that identified 38 significant SNPs on chromosome 7, the BLINK model exhibited superior stringency, potentially reflecting its enhanced ability to reduce false positives. The BLINK model demonstrated higher statistical power than the conventional MLM [22].

Furthermore, Table S3 shows that the SNP rs3469762345 explains over 70% of the phenotypic variance (PVE) for both NR and NTLV. Table S4 shows the phenotypic distributions of NR and NTLV across different rs3469762345 genotypes, revealing clear genotype–phenotype associations. On average, individuals with the GG genotype at rs3469762345 exhibited 1.22 more ribs than those with the AA genotype. The Hardy–Weinberg equilibrium test (p-HWE = 0.92) indicated no significant deviation from expected genotype frequencies, suggesting that the SNP rs3469762345 shows no evidence of being under recent selection pressure in this population. Thus, these results suggest that the SNP rs3469762345 is a valuable candidate marker for breeding schemes focused on rib number.

Three additional SNPs (rs81416674, rs81211244, and rs81347323) were identified on chromosomes other than chromosome 7 based on the BLINK model. Interestingly, in the pig QTL database, rs81347323 was associated with the feed conversion ratio [23], while rs81416674 and rs81211244 were not documented. According to the ENSEMBL database, rs81347323 is an intron variant in *PTPRT*, rs81416674 is an intron variant in *PAK1*, and rs81211244 is a synonymous variant in *ALDH7A1*.

*ALDH7A1* (Aldehyde Dehydrogenase 7 Family Member A1) encodes a key enzyme involved in lysine catabolism and cellular aldehyde detoxification. It has previously been associated with osteoporosis risk and impaired osteoblast proliferation, indicating a potential role in bone formation and skeletal integrity [24,25]. Subsequent functional studies in model organisms have provided more direct evidence linking *ALDH7A1* to skeletal development. Furthermore, targeted CRISPR-based functional screening in zebrafish has shown that the knockdown of *ALDH7A1* leads to severe skeletal deformities, underscoring its essential role in early cartilage patterning and bone fragility prevention [26]. These findings collectively suggest that *ALDH7A1* may contribute to vertebral and rib development in pigs through conserved pathways involved in cartilage matrix stability and osteogenesis. *PTPRT* (Protein Tyrosine Phosphatase Receptor Type T) is a member of the receptor-type protein tyrosine phosphatase family, which plays a key role in bone morphogenesis [27]. The *PAK1* (P21-activated kinase 1) gene has been reported to be associated with bone formation [28].

Interestingly, all of these three detected genes have been implicated in being involved in bone development, highlighting their potential as candidate genes for regulating the NTLV and NR traits in pigs. These results imply that the three newly identified SNPs could serve as valuable markers for explaining pig NTLV and NR variation. Together, these findings demonstrate that while *VRTN* remains pivotal, the novel candidate genes substantially advance our understanding of the polygenic architecture underlying porcine vertebral variation. The discovery of these new loci and genes may stem from our employment of a novel research cohort. In pig production, Landrace and Yorkshire pigs are primarily bred to develop maternal lines. Thus, our study may provide valuable insights for genetically enhancing pork carcass quality from the maternal perspective. Future functional validation in swine will be critical to unravel their contributions to vertebral and rib developmental programs.

## 4. Conclusions

In conclusion, we conducted a genome-wide association study (GWAS) to dissect the genetic architecture of the pig rib number and thoracolumbar vertebra number traits. A well-characterized QTL for vertebral number variation near the *VRTN* gene was identified, consistent with previous studies. Furthermore, three novel bone development-related variants were detected, potentially offering new insights into the genetic mechanisms underlying vertebral number plasticity.

## Figures and Tables

**Table 1:** Descriptive statistics and genetic parameters of vertebral and rib traits.

Trait	Mean	SD	Range	Genomic Variation ($\sigma_{a}^{2}$)	Heritability ($h^{2}$)
NTLV	21.3	0.581	20~23	0.223	0.700
NR	15.3	0.582	14~16	0.253	0.752
NLV	6.0	0.274	5~7	0.001	0.017

SD denotes standard deviation.

## Data Availability

Upon reasonable request, the datasets of this study can be available from the corresponding author.

## Supplementary Materials

The following supporting information can be downloaded at https://www.mdpi.com/article/10.3390/biology14081068/s1, Figure S1: 3D PCA plot. Figure S2: QQ plots for the GWAS results of the NTLV and NR traits based on the MLM and BLINK model. Table S1: Information on significant SNPs associated with NR based on the MLM analysis. Table S2: Information on significant SNPs associated with NTLV based on the MLM analysis. Table S3: Information on significant SNPs detected by BLINK model. Table S4: Phenotypic distributions of NR and NTLV across genotypes at rs3469762345.
