# Supplementary material for: A Genome-Wide Association Study of Rib Number and Thoracolumbar Vertebra Number in a Landrace × Yorkshire Crossbred Pig Population
Source: Biology (Basel). 2025 Aug 16;14(8):1068. doi: 10.3390/biology14081068 (PMC12383743; doi:10.3390/biology14081068)
Supplement: Supplementary file 1 [file biology-14-01068-s001.zip › Table S3.pdf]

Table S3 Information of significant SNPs detected by BLINK model

Chr	Pos	ID	Trait	p value	PVE* (%)	Related Genes
7	97595573	rs3469762345	NR	5.9e-67	75.26	*ABCD4~VRTN*
			NTLV	1.2e-42	70.89	
9	11850796	rs81416674	NTLV	5.9e-08	2.54	*PAK1*
2	129695476	rs81211244	NR	2.6e-07	2.28	*ALDH7A1*
17	45305224	rs81347323	NR	3.5e-08	1.57	*PTPRT*

*PVE denotes the proportion of phenotypic variation attributable to specific SNPs.
