# Supplementary material for: A Genome-Wide Association Study of Rib Number and Thoracolumbar Vertebra Number in a Landrace × Yorkshire Crossbred Pig Population
Source: Biology (Basel). 2025 Aug 16;14(8):1068. doi: 10.3390/biology14081068 (PMC12383743; doi:10.3390/biology14081068)
Supplement: Supplementary file 1 [file biology-14-01068-s001.zip › Table S4.pdf]

Table S4 Phenotypic distributions of NR and NTLV across genotypes at rs3469762345

Geno	N	NR		NTLV		*P*-HWE
		Mean	Sd	Mean	Sd	
GG	152	15.85 [a]	0.35	21.84 [a]	0.39	
GA	214	15.09 [b]	0.34	21.09 [b]	0.36	0.92
AA	73	14.63 [c]	0.49	20.67 [c]	0.47	

Class labels a, b, or c denote significant differences between genotypes at level of $p < 0.05$. Hardy-Weinberg equilibrium p-value (p-HWE) was calculated using VCFtools with the "--hardy" parameter.
