# Supplementary figures and images for: A Genome-Wide Association Study of Rib Number and Thoracolumbar Vertebra Number in a Landrace × Yorkshire Crossbred Pig Population
Source: Biology (Basel). 2025 Aug 16;14(8):1068. doi: 10.3390/biology14081068 (PMC12383743; doi:10.3390/biology14081068)

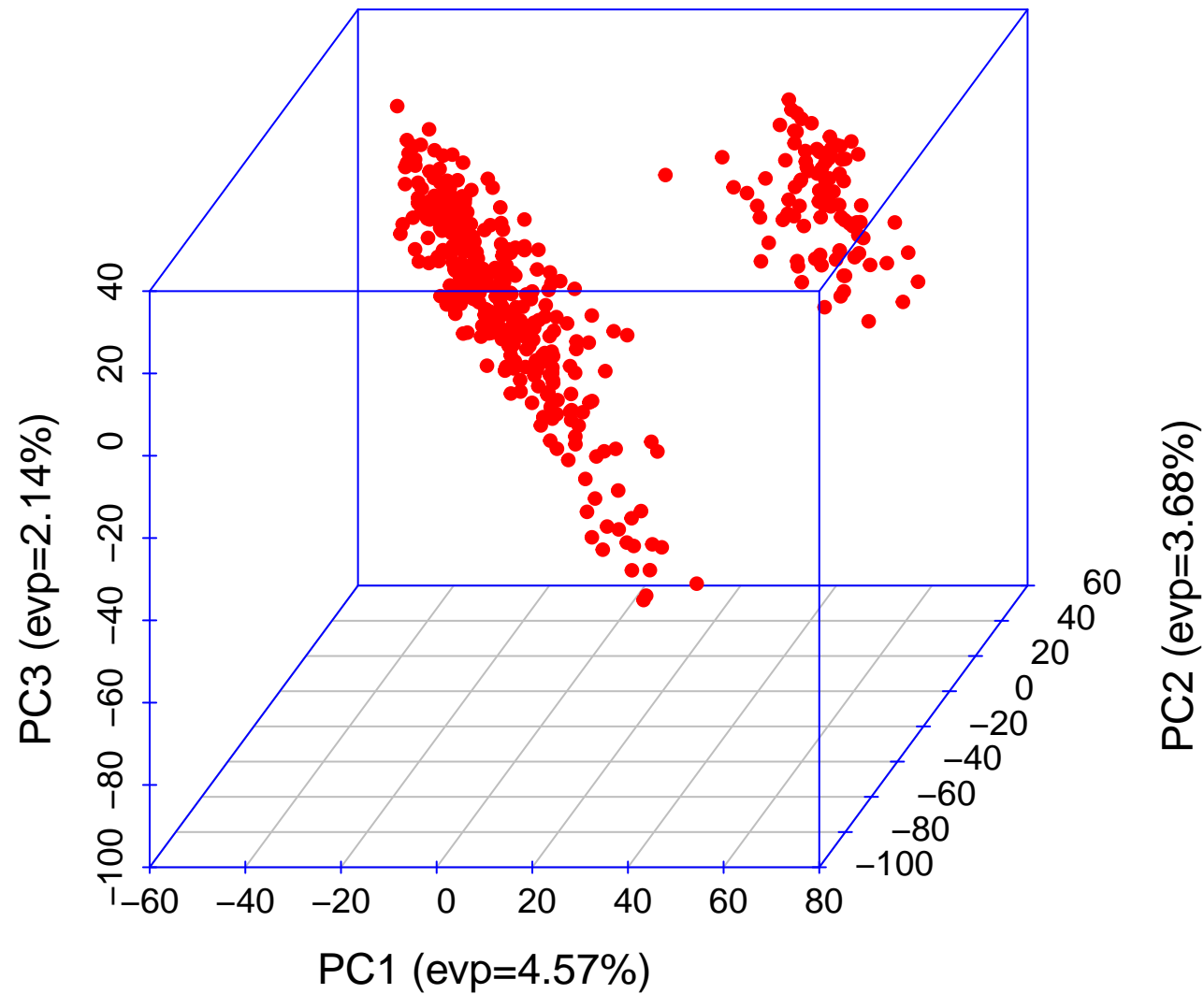

Supplement: Supplementary file 1 [file biology-14-01068-s001.zip › Figure S1.pdf]

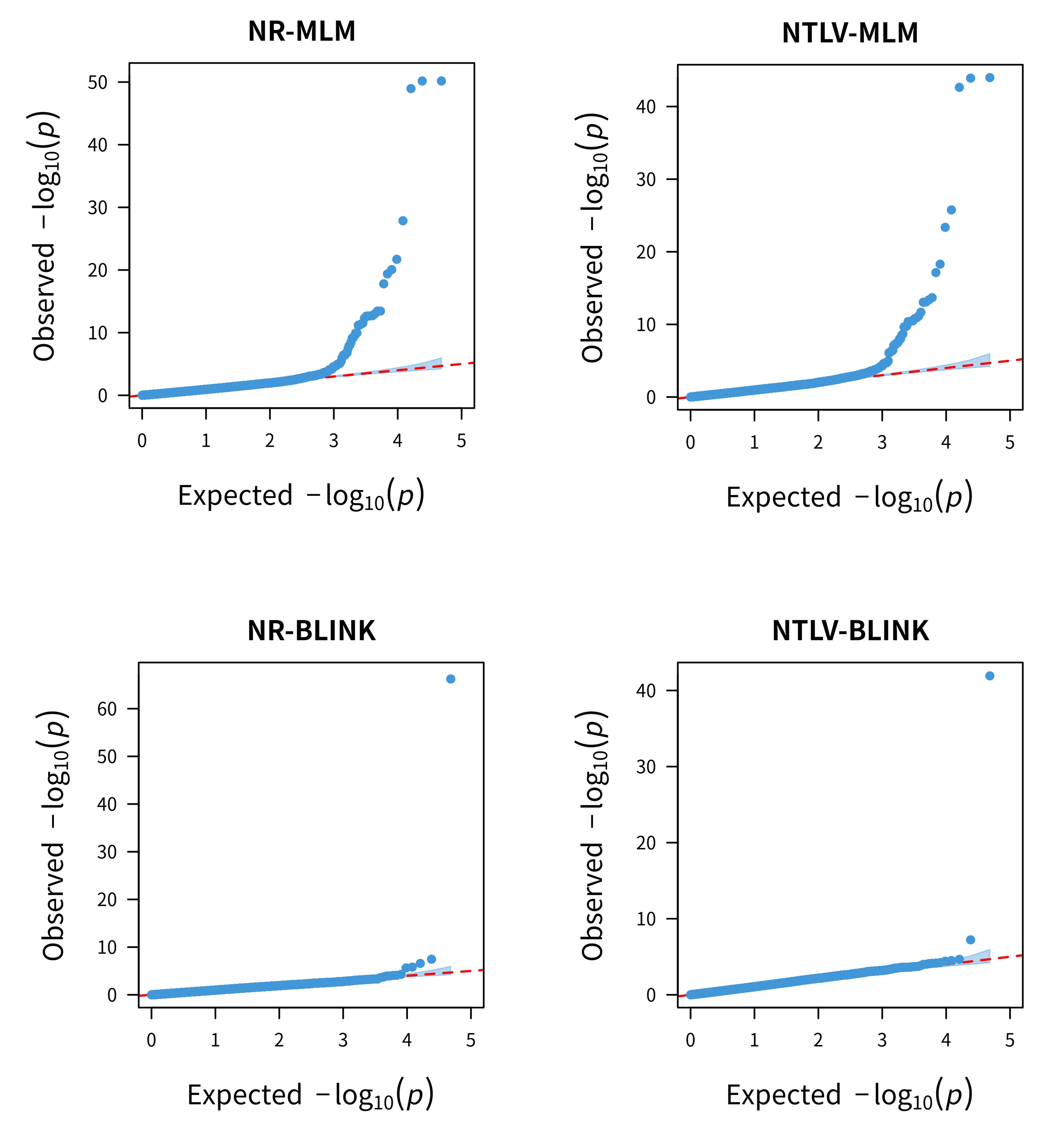

Supplement: Supplementary file 1 [file biology-14-01068-s001.zip › Figure S2.png]
